# Synaptic convergence regulates synchronization-dependent spike transfer in feedforward neural networks

**DOI:** 10.1007/s10827-017-0657-5

**Published:** 2017-09-12

**Authors:** Pachaya Sailamul, Jaeson Jang, Se-Bum Paik

**Affiliations:** 1 Department of Bio and Brain Engineering, Korea Advanced Institute of Science and Technology, Daejeon, Republic of Korea; 2 Program of Brain and Cognitive Engineering, Korea Advanced Institute of Science and Technology, Daejeon, Republic of Korea

**Keywords:** Spike transfer function, Feedforward networks, Synaptic convergence, Spike synchrony, Neural oscillation

## Abstract

Correlated neural activities such as synchronizations can significantly alter the characteristics of spike transfer between neural layers. However, it is not clear how this synchronization-dependent spike transfer can be affected by the structure of convergent feedforward wiring. To address this question, we implemented computer simulations of model neural networks: a source and a target layer connected with different types of convergent wiring rules. In the Gaussian-Gaussian (GG) model, both the connection probability and the strength are given as Gaussian distribution as a function of spatial distance. In the Uniform-Constant (UC) and Uniform-Exponential (UE) models, the connection probability density is a uniform constant within a certain range, but the connection strength is set as a constant value or an exponentially decaying function, respectively. Then we examined how the spike transfer function is modulated under these conditions, while static or synchronized input patterns were introduced to simulate different levels of feedforward spike synchronization. We observed that the synchronization-dependent modulation of the transfer function appeared noticeably different for each convergence condition. The modulation of the spike transfer function was largest in the UC model, and smallest in the UE model. Our analysis showed that this difference was induced by the different spike weight distributions that was generated from convergent synapses in each model. Our results suggest that, the structure of the feedforward convergence is a crucial factor for correlation-dependent spike control, thus must be considered important to understand the mechanism of information transfer in the brain.

**Electronic supplementary material:**

The online version of this article (10.1007/s10827-017-0657-5) contains supplementary material, which is available to authorized users.

## Introduction

Correlated neural activities are commonly found in the brain (Salinas and Sejnowski 2001). In a large neural network, mutual interaction between individual neurons often induces correlated neural activities such as periodic oscillations in firing rate, as reported in both experimental (Buzsáki et al. 1992; Buzsáki and Draguhn 2004; Courtemanche et al. 2003; Donoghue et al. 1998; Engel and Singer 2001; Klimesch 1996; Singer and Gray 1995) and computational studies (Engel and Singer 2001; Fries 2005). In general, correlated neural spike activities appear as various forms of spike synchronization (Gray and McCormick 1996; Salinas and Sejnowski 2001; Varela et al. 2001; Ward 2003) and may play an important role in the information processing in the brain (Engel et al. 2001; Fries et al. 2001; Ward 2003; Womelsdorf et al. 2007). A number of studies have reported that disruption of neural synchronization can result in a cognitive dysfunction (Başar and Güntekin 2008; Dinstein et al. 2011; Grice et al. 2001; Hammond et al. 2007; Schnitzler and Gross 2005; Uhlhaas and Singer 2006, 2010). In particular, previous studies have reported that correlated neural activities can alter spike transfer functions between neural layers (Fries 2005, 2009; Ratté et al. 2013; Wang et al. 2010), implying that varying spike transfer may play a role in modulating the dynamics of a neural network. However, it is not clear yet, if this synchronization-dependent spike transfer, which might work as a mechanism of dynamic control of spike transfer, can be achieved conditionally on a specific feedforward pathway structure, or rather, be achieved independent of underlying circuitry. Here we address this question by performing computer simulations of feedforward neural networks with different types of convergent synaptic connections.

A feedforward network is generally composed of unidirectional interlayer connections from the lower (source) to the higher (target) level neural layers (Felleman and Van Essen 1991; Kumar et al. 2010). In most cases, each cell in the target layer receives input from more than one source cell through convergent synaptic connections, as observed in the thalamocortical connections in the visual system (Hubel and Wiesel 1962). Here we hypothesize that the achievement of synchronization-dependence of spike transfer is reliant on convergent wiring in the feedforward pathway.

To test our idea, we developed a model simulation of three convergence rules that have different spatial distributions of synaptic connection probability and strength. This included one where the connection probability and the strength were independent of the spatial distance between source and target cells, and one where all the connection parameters systemically changed as a function of neural distance. Then we examined how the spike transfer function changes under these conditions, while we varied the synchronization level of input spikes.

We first confirmed that the spike transfer function of the model neural network alters depending on the level of input synchronization. In addition, we found that the modulation of spike transfer function strongly depends on the convergence-wiring rule, because the synchronization-dependency of transfer function appeared significantly different in each convergence model. We observed that the spike transfer function of the target neuron was sensitively altered by the convergence structure, because the weight distribution of input spikes were significantly different in each convergence condition, even for identical input sources.

This result suggests that feedforward convergence is a crucial factor for achieving the correlation-dependent spike transfer in neural systems, and may provide insight about the mechanism of information processing in the brain.

## Materials and methods

### Development of cell mosaics

**Fig. 1:**
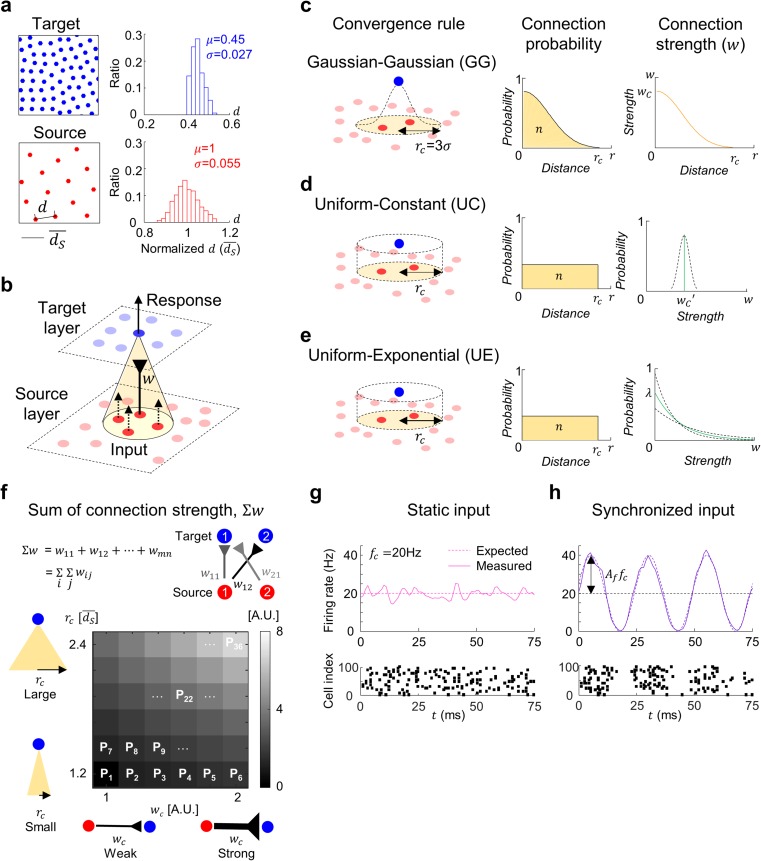
Different models of convergence in feedforward neural networks. (**a**) Part of source and target layer of feedforward network and its distribution of nearest neighbor distance (*d*); $\overline{d_S}$ is the expected unit distance between two cells in the source layer when every cell is distributed in a perfectly hexagonal lattice pattern. Each layer was developed from a revised PIPP model. The terms *μ* and *σ* are the average and standard deviation of the nearest neighbor distance of the mosaic structure, respectively. (**b**) Structure of a feedforward neural network. (**c–e**) Three different convergence rules. A target cell samples convergent input from source cells in range (*r*_*c*_) with synaptic connection strength (*w*). Estimated number of total synaptic connections (*n*) and sum of connection strength (*Σw*) of a target cell were set to be equal between models. **(c)** Gaussian-Gaussian (GG) model: Both the connection probability and the strength are given as Gaussian distribution as a function of distance between source and target cells. (**d–e**) Uniform-Constant (UC) and Uniform-Exponential (UE) models: The connection probability is uniform within a certain range and the connection strength is set as a constant (UC) or an exponentially decaying function (UE), respectively. The dotted lines indicate the range of allowed variation of connection strength across target neurons. (**f**) Sum of connection strength (*Σw*) for different conditions of convergence: *Σw* increases as *r*_*c*_ or *w*_*c*_ increases. P_22_ was selected for further analysis. (**g-h**) Amplitude of oscillation in mean firing rate of source neurons modulates the level of synchronization in source activity: **(g)** Static (*A*_*f*_ = 0) input generated by source cells, **(h)** Synchronized input (*A*_*f*_ = 1). *Top*, Instantaneous firing rate of source cells. *Bottom*, Raster plot of spikes in 100 source cells. Synchronization in spike timings is observed for synchronized input

To decide the spatial distribution of cells in source and target layers, we used an adapted version of a pairwise interaction point process (PIPP) model, which is a computational model of cell mosaic development (Eglen et al. 2005), where each cell is relocated until the new position satisfies the designed mosaic statistics (Fig. 1a,b). As a modification of the PIPP model, we introduced a local repulsive interaction between the nearby cells that induces a gradual shift of each cell position. Source and target layers were developed independently from an initial random distribution of cells. To avoid the sampling bias in the boundary area, the target layer was designed smaller than the source layer, with different unit distances for source (*d*_*s*_) and target (*d*_*T*_) layers, $\overline{d_S}=2.2\overline{d_T}$.

For the local repulsive interaction, we used a sigmoidal function $\overrightarrow{F}\left(\overrightarrow{r}\right)$, so that the strength of repulsion increases as two cells at ${\overrightarrow{x}}_1$ and ${\overrightarrow{x}}_2$ get closer.

$$ \overrightarrow{F}\left(\overrightarrow{r}\right)=\left\{\begin{array}{c}\hfill \frac{a}{1-\exp \left[-{\left\{\left(\left|\overrightarrow{r}\right|-\delta \right)/\phi \right\}}^{\alpha}\right]}\overrightarrow{u}\kern1.25em \left(\delta <\left|\overrightarrow{r}\right|<2\overline{d}\right)\hfill \\ {}\hfill \kern6em 0\kern8.5em (otherwise)\hfill \end{array}\right. $$

where $\overrightarrow{r}={\overrightarrow{x}}_1-{\overrightarrow{x}}_2$, the coefficient *a* = 10^−5^, $\overrightarrow{u}=\frac{\overrightarrow{r}}{\left|\overrightarrow{r}\right|}$ and the parameters for sigmoidal function were *α*=1.6, *ϕ*=5.7$\overline{d}$ and *δ*=0.089$\overline{d}$.

At each time *t*, velocity of a cell ${\overrightarrow{v}}_t$ is decided by the sum of all repulsive interactions between the target and other cells, ${\overrightarrow{F}}_{net,t}$.

$$ {\overrightarrow{v}}_t={\overrightarrow{v}}_{t-1}+{\overrightarrow{F}}_{net,t} $$

$$ {\overrightarrow{F}}_{net,t}=\sum_i{\overrightarrow{F}}_{i,t}-c\left|{\overrightarrow{v}}_{t-1}\right|\times {\overrightarrow{v}}_{t-1} $$

To prevent too fast movement of cells, $c\left|{\overrightarrow{v}}_{t-1}\right|\times {\overrightarrow{v}}_{t-1}$ was added as a friction term, where *c* = 0.1. We allowed 5000 iterations for the development of each mosaic.

### Single model neuron

We developed a single model neuron in the target layer using NEURON simulator (Carnevale and Hines 2006), based on the Hodgkin-Huxley model (Hodgkin and Huxley 1952) as:

$$ C\frac{dv}{dt}=-{g}_L\left(v-{V}_L\right)-{G}_{Na}\left(v-{V}_{Na}\right)-{G}_K\left(v-{V}_K\right)-{G}_{CaT}\left(v-{V}_{CaT}\right)-{g}_E(t)\left(v-{V}_E\right) $$

where *C* is membrane capacitance, *g*_*L*_ is leakage conductance, *g*_*E*_ is excitatory synaptic conductance from input spikes, *G*_*X*_ is conductance for X ion channel, and *V*_*X*_ is reversal potential for the X ion channel. We included a sodium channel (*Na*), a potassium channel (*K*), a T-type calcium channel (*CaT*), and an excitatory synaptic input channel (*E*). The ion channel conductance terms *G*_*Na*_, *G*_*K*_, and *G*_*CaT*_ are functions of membrane potential *v*, and take the general form as in previous studies (Hodgkin and Huxley 1952). The parameter values were determined from previous studies (Hodgkin and Huxley 1952), as *G*_*Na*_ = 120 mS/cm^2^, *G*_*K*_ = 36 mS/cm^2^, *G*_*L*_ = 0.4 mS/cm^2^, *G*_*CaT*_ = 2 mS/cm^2^, *E*_*Na*_ = 55 mV, *E*_*K*_ = −80 mV, *E*_*L*_ = −65 mV, *E*_*CaT*_ = 126.1 mV . A single neuron was designed as a point model of cylindrical shape with both height and diameter equal to 28 μm. The membrane capacitance was set to 1 μF/cm^2^ and resistance to 200 ohm· cm. All the synaptic interactions, or excitatory postsynaptic conductance (EPSC) were modeled as two-parameter alpha function, *g*_*E*_(*t*) = *w* · [exp(−*t*/*τ*_2_) − exp(−*t*/*τ*_1_)], where *τ*_1_= 1 ms and *τ*_2_= 3 ms are the rise and decay time constants, and *w* is a synaptic-strength weight factor (Carnevale and Hines 2006). The activities of neurons in the network were simulated for five seconds in each trial, and were repeated for 10 trials.

### Model feedforward networks of different convergence rules

To build up a simple feedforward network, we first developed cell mosaics of source and target layers of network, using an adapted version of a pairwise interaction point process (PIPP) model (Eglen et al. 2005), which assumes a local repulsive interaction between neighboring cells (Fig. 1a, see Methods 2.1 for detailed model design). The source and target layers included 1150 and 166 cells, respectively. For simplicity, all the neurons were assumed to be excitatory and all the spatial length units were normalized with the expected unit distance ($\overline{d_S}$) between two cells in the source layer when every cell is distributed in a perfectly hexagonal lattice pattern. To develop various convergent connections between the source and target layers (Fig. 1b), we designed three wiring rules that consider only two variables: connection probability and connection strength (Fig. 1c,d, and e).

First, in the Gaussian-Gaussian (GG) model, which is conventionally used for inter-neural connectivity in network model studies (Paik et al. 2009; Paik and Glaser 2010; Ringach 2004), the synaptic connection probability and connection strength follow the 2D Gaussian as a function of distance between the source and target cells: $y=A\ \mathit{\exp}\left(-\frac{d^2}{2{\sigma}^2}\right)$, where *d* is distance between cells, *A* is the maximum probability at *d* = 0, and *σ* is the standard deviation, which controls the width of the Gaussian curve (Fig. 1c, *left*). For connection probability in our model, *A* was set to 0.85 within the range of connection *r*_*c*_= 3*σ*, and to 0 outside this range (McLaughlin et al. 2000; Reid and Alonso 1995). Under this condition, we could estimate the expected number of convergent connections per target neuron, *n*, from the area below the connection probability distribution (Fig. 1c, *middle*). Using this number *n* in the GG model, we made a calibration between convergence models so that the actual number of connections was about the same in all models. Next, the connection strength for the GG model was defined similarly, by the same Gaussian function where the maximum strength of connection *w*_*c*_ was set as a variable to determine the strength level of synaptic connections (Fig. 1c, *right*).

For the other two convergence rules, Uniform-Constant (UC) and Uniform-Exponential (UE) models, the connection probability does not depend on the distance between the source and target cells. This connection probability is set as a uniform distribution within the range *r*_*c*_ estimated in the GG model (Fig. 1d and e, *middle*), and the constant value of this connection probability is calculated so that the expected number of connections per target neuron becomes the same as *n* in the GG model.

The connection strength for each target cell in the UC and UE models were also normalized so that the expected value of total connection strength ( ∑ *w*) for each target cell was the same in all models. In the UC model, the connection strength of every synapse connected to a specific target cell was set to a constant, *w*_*c*_^'^ (Fig. 1d, *right*), which is the mean of connection strength connected to that target cell in the GG model. In the UE model, which is based on the observation of thalamocortical circuits (Jin et al. 2011), the strength of each connection was randomly sampled from an exponentially decaying probability distribution, *p*(*w*) = *λ* exp(−*λw*), independent of the distance between the source and target cell (Fig. 1e, *right*). Also in this case, the value of *λ* was properly chosen so that the sum of connection strength was always equal to that in GG model. Due to the stochastic process in connection wiring, the value of *w*_*c*_^'^ and *λ* slightly vary for each target cell.

### Variation of convergence parameters and total synaptic weight

In our convergence models described above, the sum of all convergent synaptic connection weight for a target neuron can be represented by two parameters in the GG model: range of connection, *r*_*c*_ and strength of connection, *w*_*c*_ (Fig. 1c). This is because the other two models are set to have the same amount of total synaptic weight as the GG model. In general, *r*_*c*_ decides how many source cells will be connected to a target cell, and *w*_*c*_ determines how strong the connections will be. As each parameter increases, the total feedforward connection strength ∑*w* increases. To test various cases of convergence parameters and connection strength ∑*w* within each model, we simulated the activity of a model network for 36 parameter sets (P_1_–P_36_, Fig. 1f).

### Static and synchronized input spike patterns

To simulate different conditions of input spike correlation, two types of input pattern (static and synchronized (Sync) inputs) were designed to provide a source activity for the feedforward network (Fig. 1g, h). The static input pattern of a source cell was generated by a Poisson spike generator with constant mean firing rate, *f*_*c*_= 20 Hz (Fig. 1g, *top*). On the other hand, for synchronized input, mean firing rate *f*(*t*) of the spike generator of each cell was given as a sinusoidal function (Fig. 1h, *top*) as

$$ f(t)={f}_c\left(1+{A}_f\sin \left(2\pi {f}_{OSC}t\right)\right) $$

Because the phase of the oscillation is identical for all the source neurons, this oscillation induces synchronized activity in the input spikes of source neurons (Fig. 1g, h, *bottom*). For the synchronized input pattern, we simulated different levels of oscillation by varying *A*_*f*_ from 0 (no synchronization, or static; Fig. 1g) to 1 (Strong synchronization; Fig. 1h). The frequency of oscillation *f*_*osc*_ was set to 40 Hz to model a gamma band oscillation.

## Results

### Synchronization-dependent response modulation in a feedforward network

We investigated the synchronization-dependent spike transfer of the three different convergent feedforward model networks. We measured the response of target neurons while varying the spike patterns of the source neurons, from static to strongly synchronized (*A*_*f*_= 0 to 1), as different levels of synchronized input.

To investigate the response activity of the target layer, the “spike transfer function” of a network was estimated by measuring the firing rate of the target layer as the response (*R*) to a given input of fixed spike counts. Furthermore, “synchronization-dependent spike transfer” was defined as the ratio between spike transfer for synchronized input and for static input (*R*_Sync_/*R*_Static_), to examine the effect of input synchronization more precisely.

**Fig. 2:**
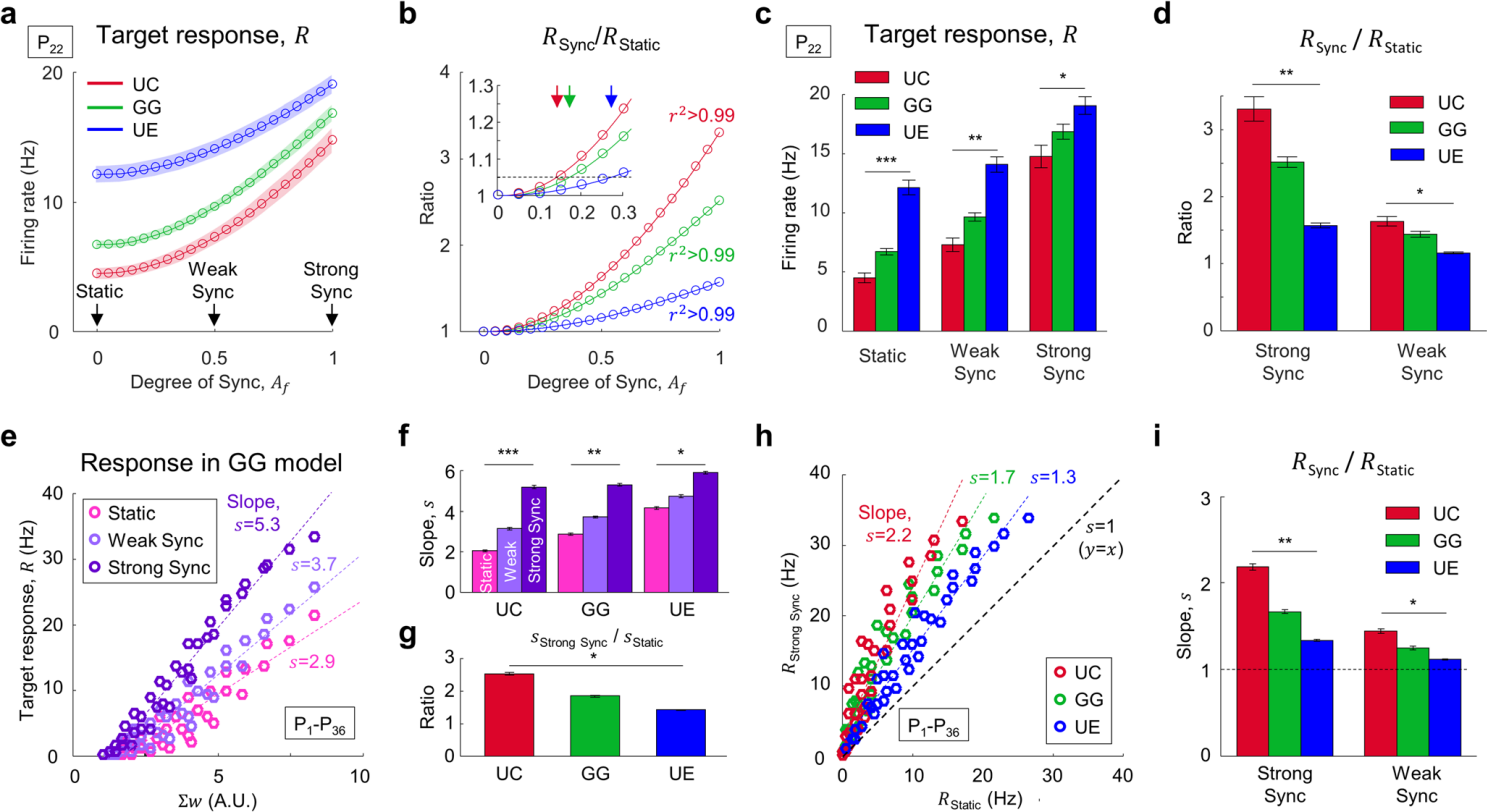
Response firing rate increases as input is synchronized, with the greatest increase in UC model. (**a**) Response (*R*) of target neurons for different degree of synchronization in the activity of source neurons. The response increases as *A*_*f*_ increases. *A*_*f*_= 0, 0.5, 1 are chosen for further analysis. Shaded area indicates standard variation. (**b**) Response for different degree of synchronization normalized by the response for static input (*A*_*f*_ = 0). As *A*_*f*_ increases, the response increases most in the UC model. The average of the ratio at *A*_*f*_ = 1 is 3.31, 2.51, and 1.57; in the UC, GG, and UE model, respectively. Each curve is fitted to a cubic polynomial and *r* ^2^ > 0.99 in all the models. In the magnified inset, arrows indicate the threshold of *A*_*f*_, at which more response is induced than by static input, where each curve exceeds 5% above ‘1’. Threshold is at *A*_*f*_ = 0.142, 0.173, and 0.272; in the UC, GG, and UE model, respectively. (**c**) Response (*R*) of target neurons for a particular set of parameter condition, P_22_ (One-way ANOVA followed by *post hoc* Bonferroni analysis, * *p =* 5.7×10^−6^, ** *p =* 9.2×10^−10^, *** *p =* 1.1×10^−10^). (**d**) Ratio of *R* for strongly/weakly synchronized and static input (One-way ANOVA followed by *post hoc* Bonferroni analysis, * *p =* 4.8×10^−9^, ** *p =* 1.7×10^−14^). Change of *R* appears most significant in the UC model. **(e)** Response of GG model for static and synchronized (weak: *A*_*f*_= 0.5, strong: 1) input. Pearson correlation coefficient (*r*) and *p*-value of linear fitting for every input pattern are shown. (Pearson correlation coefficient, *r* = 0.94, 0.96, 0.98 and *p =* 2.2×10^−16^, 2.3×10^−20^, 2.2×10^−26^ for static, weakly and strongly synchronized input, respectively) Responses of the UC and UE models are not shown. **(f)** Slope between response and *Σw* in each model (One-way ANOVA followed by *post hoc* Bonferroni analysis, * *p =* 7.2×10^−17^, ** *p =* 1.0×10^−22^, *** *p =* 1.4×10^−24^). In every model, slope increases as the input pattern is better synchronized. **(g)** Ratio of slope for strong synchronization over static input in each model (One-way ANOVA followed by *post hoc* Bonferroni analysis, * *p =* 4.2×10^−22^). Increase of slope for synchronized input is greatest in the UC model. **(h)** Responses of three models for static and strongly synchronized input. Pearson correlation coefficient (*r*) and *p*-value of linear fitting are shown for every model. (Pearson correlation coefficient *r* = 0.95, 0.95, 0.95 and *p =* 1.6×10^−17^, 1.9×10^−19^, 3.6×10^−23^ for UC, GG, UE model, respectively) The plot between static and weakly synchronized input is not shown. **(i)** Slope between response for strongly/weakly synchronized input and static input (One-way ANOVA followed by *post hoc* Bonferroni analysis, * *p* = 3.9×10^−14^, ** *p* = 2.4×10^−21^). The ratio of response for synchronized input over response for static input is greatest in the UC model

First, we compared the spike transfer between the convergence models. For a fixed parameter set, we observed that spike transfer increased as the input synchronization became stronger (Fig. 2a). Interestingly, we noted that the mean firing rate of the target neurons appeared different across the convergence models for each type of given input. Because our main interest was not the spike transfer itself, but how much the response increased for the synchronized input, we measured the “synchronization-dependent spike transfer”, the ratio between the responses for static and synchronized input patterns (*R*_Sync_/*R*_Static_) (Fig. 2b). Although the response itself was greatest in the UE model for all the input, the ratio of increase of firing rate for synchronized input was greatest in the UC model. For further statistical analysis, we selected three conditions of synchronization *A*_*f*_= 0 (static), 0.5 (weak Sync), 1 (strong Sync), and confirmed that both the mean firing rate and the ratio between the static and synchronized conditions were significantly different across convergence models in all cases (* *p =* 5.7×10^−6^, ** *p =* 9.2×10^−10^, *** *p =* 1.1×10^−10^, one-way ANOVA followed by *post hoc* Bonferroni analysis; Fig. 2c and * *p =* 4.8×10^−9^, ** *p =* 1.7×10^−14^, one-way ANOVA followed by *post hoc* Bonferroni analysis; Fig. 2d).

Next, to confirm the difference between the models for the other convergence conditions, we observed that the mean firing rate of target neurons increased as the total feedforward connection strength (*Σw*) increased in the 36 different conditions of parameter sets (P_1_-P_36_) we tested (Fig. 2e). This relationship between the sum of synaptic weight *Σw* and the firing rate of response was well fitted to a linear function. We found that the slope of this linear fit noticeably varied as we varied the input spike correlation from static to synchronized patterns (Fig. 2e). In all three convergence models (GG, UC, and UE), the slope increased as the correlation level in the input increased (* *p =* 7.2×10^−17^, ** *p =* 1.0×10^−22^, *** *p =* 1.4×10^−24^, one-way ANOVA followed by *post hoc* Bonferroni analysis; Fig. 2f). This result confirms that synchronized or temporally correlated inputs can transfer more spikes than uncorrelated inputs in a feedforward network. In other words, even when the number of input spikes is the same, the number of transferred spikes can significantly vary depending on the level of input synchronization. This suggests a synchronization-dependent modulation of spike transfer. Interestingly, we observed that the modulation of spike transfer by input correlation appeared different across the convergence models (* *p =* 4.2×10^−22^, one-way ANOVA followed by *post hoc* Bonferroni analysis; Fig. 2g). We found that the change of the slope in the response function induced by input synchronization was significantly larger in the UC convergence model than in the GG or UE models. In other words, the spike-transfer function of the network with UC-type convergence was more susceptible to the change of synchronization level than that with the other two convergence types.

To examine this further, we compared the response firing rates of the system to static input and to strongly synchronized input, for each condition of *Σw* (Fig. 2h). We confirmed that the response activity to synchronized input was always higher than that to static input, because the slope of the Response(Static) *vs.* Response(Sync) graph was always greater than 1. More importantly, we found that this slope is larger in UC model than in the other two models for both weak and strong synchronization-input conditions (* *p* = 3.9×10^−14^, ** *p* = 2.4×10^−21^, one-way ANOVA followed by *post hoc* Bonferroni analysis; Fig. 2i). This result shows that the modulation of spike transfer by synchronization is the most significant in UC-type convergence, and suggests that the structure of the feedforward convergence is a critical factor for achieving a synchronization-dependent spike transfer.

We performed additional simulations to investigate the effect of heterogeneous oscillation phase of each individual source neuron activity (Supplementary Fig. S1). We observed, in all three models, that the response increased as the oscillating phase of each individual neuron was more sharply synchronized, similar to the result where synchronization was modulated by the amplitude of oscillation (Fig. 2a, b). We were also able to estimate the response dependence on the phase synchronization by calculating the response change ratio (Supplementary Fig. S1c). Again, the response of the UC model was most dependent on the degree of phase synchronization, similar to the oscillation strength dependence in Fig. 2.

### Synchronization-dependent spike transfer for a single spike input

**Fig. 3:**
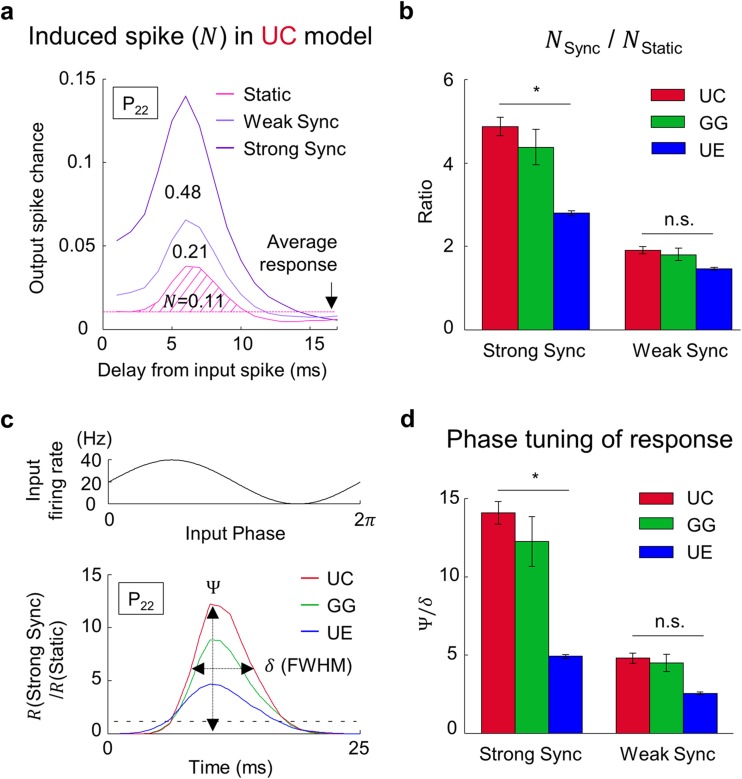
Synchronization-dependent response is greatest in the UC model. **(a)** The number of average target spikes (*N*) induced by one source spike for static and synchronized input pattern in UC model at P_22_. *N* is counted by excess target spike activity over the mean of the output-spike probability (dashed line). (**b**) Ratio of *N* between strongly/weakly synchronized and static input (One-way ANOVA followed by *post hoc* Bonferroni analysis, * *p* = 1.4×10^−3^, n.s. *p* = 0.094). Increase of *N* for synchronized input is greatest in the UC model. **(c)** Phase-dependent spike transfer in each cycle of input oscillation. Variation of response for strong synchronization is normalized by response for static input during one period of oscillation at P_22_. Ψ is amplitude and *δ* is full width at half maximum of tuning curve. Dotted line at ‘1’ indicates the average response level for static input. **(d)** Phase tuning between output response and oscillation of input spike pattern (Ψ/*δ*) (One-way ANOVA followed by *post hoc* Bonferroni analysis, * *p* = 1.3×10^−3^, n.s. *p* = 0.26). The degree of tuning between input and output for strong synchronization is greatest in the UC model

Next, we tested to see if the convergence-dependent modulation of the spike transfer is also observed in response to a single spike input. In each simulation condition above, we examined the average response per single input spike in each neuron (Fig. 3a). Specifically, in each target neuron, we estimated the average number of induced spikes (*N*) after every single input spike was received. Here, *N* is defined as the area above the mean response level in the histogram of output spikes, after each input spike to the cell (Fig. 3a). For example, in the UC model at P_22_, *N* = 0.11 for static input, and *N* = 0.48 for strong synchronization. To investigate the dependency on the input synchronization, the ratio of *N* between synchronized and static input patterns are compared in each model (* *p* = 1.4×10^−3^, n.s. *p* = 0.094, one-way ANOVA followed by *post hoc* Bonferroni analysis; Fig. 3b). We found that this ratio was highest in the UC model and that the ratios of different convergence models were statistically distinguishable.

This result reveals that every single input spike may have a different probability of inducing a spike response, depending on change in the correlation level of inputs. Moreover, this correlation-dependent response modulation appeared strongest in UC-type feedforward convergence, compared to UE and GG types. From these results, we confirmed that the synchronization-dependent spike transfer is most significant in the UC model, from both population and single-spike level analysis.

Although N was different across static and synchronized input, the peak of output spike chance was consistently at 6 ms of delay from an input spike for different levels of synchronization. Considering that an identical set of rise and decay time constants in EPSC was used in all models, the length of delay is expected to mainly depend on the form of EPSC, rather than the degree of oscillation or the convergence structure. The dynamics of delay was not investigated further, because our main interest was the varying part of the network originated by the different convergence structures.

### Oscillation phase-dependent spike transfer

Next, to test if the convergence-dependent response modulation we found could be instantaneously controlled by the temporal correlation of input spikes, we investigated the phase-dependency of spike transfer in each cycle of input oscillation (Fig. 3c). In a period of input oscillation (1/40 Hz = 25 ms), we counted the average number of induced spikes as a function of the oscillation phase (colored solid lines), and compared this with the spike responses to static input (black dashed line). We observed that the spike responses in synchronized inputs are phase-locked to different degrees, depending on the oscillation strength in all convergence conditions. To analyze this quantitatively, we measured the amplitude (Ψ) and the width (δ, full width at half maximum) of the spike response curve and calculated Ψ/δ as the index of sharpness of phase tuning. We found that the value of Ψ/δ appears noticeably different across convergence types, and is higher in UC model than in the other two (* *p* = 1.3×10^−3^, n.s. *p* = 0.26, one-way ANOVA followed by *post hoc* Bonferroni analysis; Fig. 3d), suggesting that UC-type convergence can best perform a synchronization-dependent spike filter, or transfer control, among the models we tested.

Our results show that the types of feedforward convergence circuits may determine the effectiveness of synchronized input spikes in a way that the network becomes either a very dynamic synchronization-dependent spike filter, or just a robust relay station that is independent of input spike correlation. Among those convergence models we tested, we found that UC-type feedforward convergence could work as an effective control of spike transfer that modulates spike transfer depending on the instantaneous change of spike correlation in the input pattern.

### Convergence structure regulates input spike profiles towards a target cell

Having observed that the spike transfer function and the synchronization dependency of each model network varied significantly, we then examined whether this observed difference between the models could be explained by their feedforward convergence circuit structures.

**Fig. 4:**
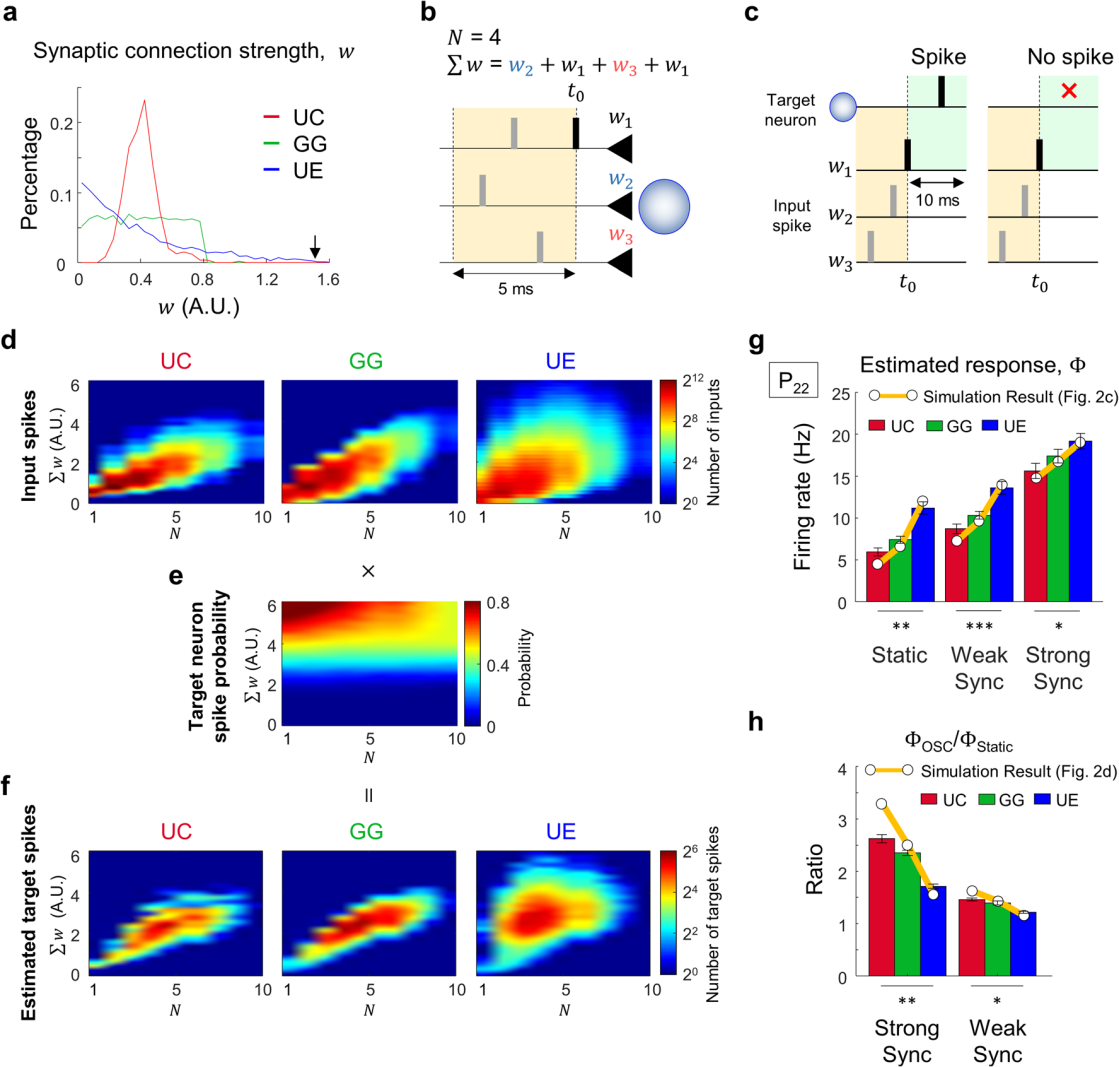
Distribution of connection strength regulates response sensitivity to input synchronization. (**a**) Averaged distribution of connection strength (*w*) of all input synapses onto a target neuron in each model. Black arrow indicates *w* = 1.51, the threshold that a single input spike can generate a target spike **(b)** Example of input profile parameters (*N*, ∑*w*) within a temporal window. In the spike trains generated by source cells connected to a common target cell, the number of input spikes (*N*) and connection-weight sum (∑*w*) of input spikes within a temporal window (5 ms) were measured before every input spike. **(c)** Each input pattern of parameters (*N*, ∑*w*) was examined to see if it induced a spike in a target cell or not, within 10-ms after each onset spike. To avoid over-counting the number of windows that induced a target spike, only the cases that did not have any additional input spike between the onset and the target spike was counted. (**d**) Distribution of the parameter sets, (*N*, ∑*w*), of all input windows for static input. The number of inputs of parameter (*N*, ∑*w*) was counted for each model. (**e**) Averaged target spike probability of all three models combined. See Supplementary Fig. S2 for details **(f)** Estimated target response in each model obtained by multiplying the input spike distribution in **(d)** and the target spike probability in **(e)**. **(g)** Target cell firing (Φ) was estimated by summing all the response matrix components in **(f)**. They appeared significantly different across models (One-way ANOVA followed by *post hoc* Bonferroni analysis, * *p* = 3.7×10^−4^, ** *p* = 1.1×10^−5^, *** *p* = 8.9×10^−6^), and the difference was consistent with the result simulated in Fig. 2c **(h)** The Φ ratios of static to synchronized input patterns were significantly different across models, corresponding to the observed result in Fig. 2d (One-way ANOVA followed by *post hoc* Bonferroni analysis, * *p* = 1.8×10^−4^, ** *p* = 2.5×10^−10^). For **(g)–**(**h)**, the observed results in Fig. 2c-d were indicated as orange solid lines for comparison

In our model network, we confirmed that the distributions of individual connection weights toward a target cell were noticeably different across models (Fig. 4a), even though the total synaptic connection weights were set to be consistent in all models (area under each plot, Fig. 4a). Thus, we expected these disparities would induce different input spike profiles for each target neuron and result in dissimilar target cell activities.

To investigate how the identical source neuron activity is converted into different input patterns for a target cell by each convergence structure, we measured the number (*N*) and the connection-weight sum (∑*w*) of input spikes within a temporal window (5 ms) before every input spike (*t*_0_, Fig. 4b) that a target cell received. We assumed that the strength and synchrony of input spikes can be simply described with these two parameters (*N* ,  ∑ *w*). Based on this assumption, we determined whether each input pattern of a parameter set (*N* ,  ∑ *w*) could induce a spike in a target cell after each onset spike (Fig. 4c). As a result, we observed that the 2D profiles of input patterns appear noticeably different across convergence types (Fig. 4d), indicating that different synaptic convergence conditions induce dissimilar input spike patterns onto a target cell, even from identical input sources.

On the other hand, the spike probability for each condition of (*N*, ∑*w*) appears fairly similar across models (Fig. 4e, See Supplementary Fig. S2 for details). This is understandable because this result is dependent on the target cell response function only, which is identical in all models. This observation shows that we can use the same spike probability function to estimate output activity level for all models, for a given input profile. Using this result, we evaluated the expected number of target spikes (Fig. 4f) by multiplying the target spike probability (Fig. 4e) and the measured input profile of (*N*, ∑*w*) in each model (Fig. 4d). Then, we compared the estimated target cell firing, Φ with the simulated result in Fig. 2c, d. The estimated response, Φ, in the UC, GG, and UE models for both static and synchronized input patterns were noticeably different from each other (* *p* = 3.7×10^−4^, ** *p* = 1.1×10^−5^, *** *p* = 8.9×10^−6^, one-way ANOVA followed by *post hoc* Bonferroni analysis; Fig. 4g), and well agreed with the observed result in Fig. 2c. In addition, we calculated the expected synchronization-dependency of network activities, from the Φ ratio for static and synchronized input patterns in each model. The observed result showed significant difference across the models (* *p* = 1.8×10^−4^, ** *p* = 2.5×10^−10^, one-way ANOVA followed by *post hoc* Bonferroni analysis; Fig. 4h), and agreed fairly well with the simulation result in Fig. 2d. This indicated that the convergence-dependent input pattern variation could explain the dissimilar synchronization-dependency character between the models.

### Functional implications across different convergence types and ranges

**Fig. 5:**
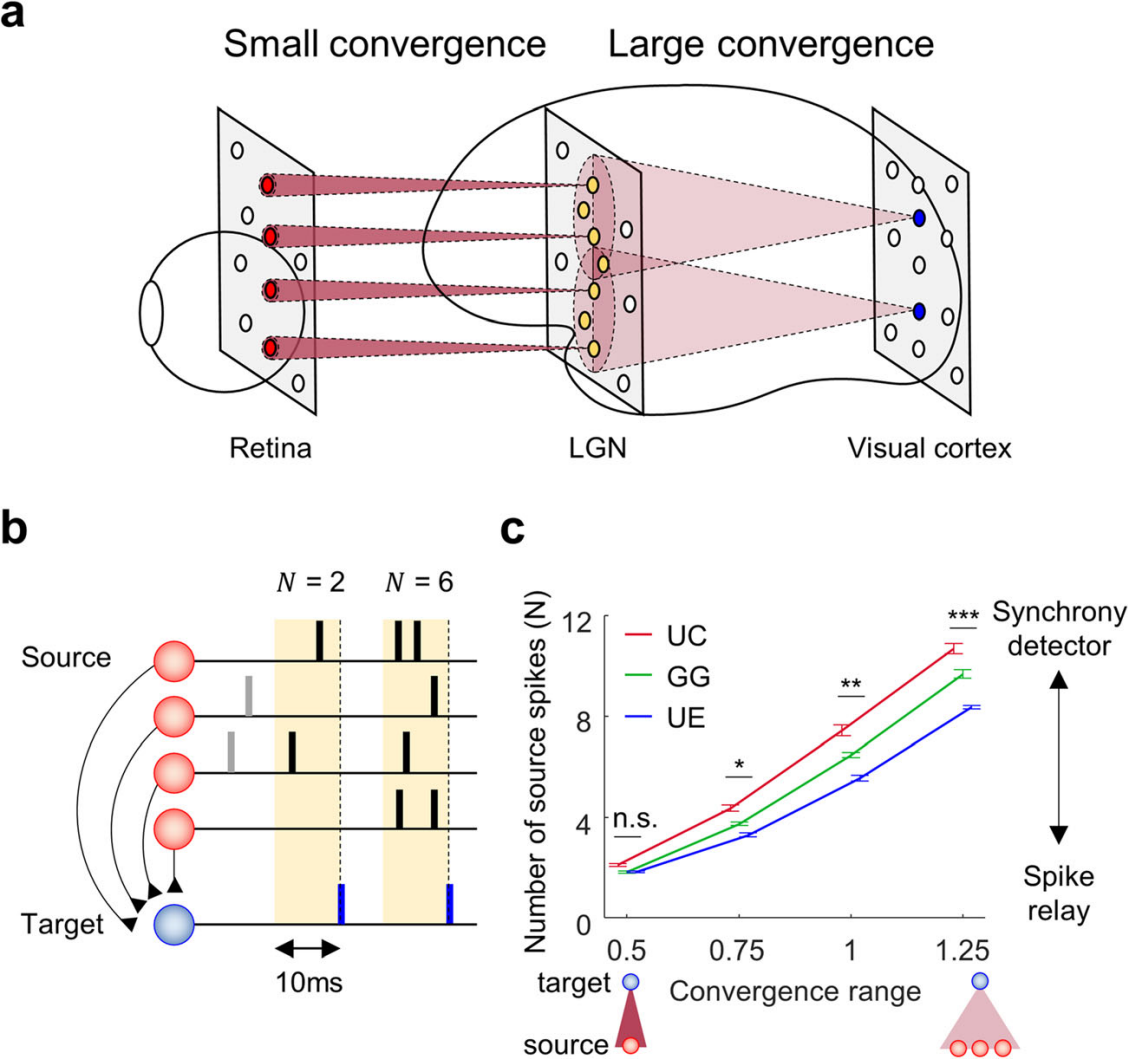
Network with large convergence sensitively responds to input synchronization, while network with small convergence stably relays source activity. (**a**) Illustration of convergence circuits in visual pathway; a small convergence between the retina and the LGN, and a large convergence between the LGN and the visual cortex. (**b**) Characteristic of spike transfer. The number of source spikes (N) within 10 ms before a target spike indicates how many source spikes are required to produce a target spike. Larger N implies that more spikes are needed to provide a target spike. (**c**) The number of source spikes N across different conditions of convergence. As the range becomes larger, N in each model generally increases, implying that the network needs more input spikes within a short time to generate a target spike. This condition makes the network with large convergence respond only to synchronized inputs, as a coincidence detector. Note that the difference between the models becomes larger as convergence range increases. This implies that spike transfer function of a large convergence strongly depends on the structure of convergence circuits. (One-way ANOVA followed by *post hoc* Bonferroni analysis, * *p* = 2.2×10^−10^, ** *p* = 2.4×10^−13^, *** *p* = 8.3×10^−14^, n.s. *p* = 1). The average number of connections for a target cell at each range is 4.4, 9.8, 17.5, and 27.2, respectively

In the neural system, it has been observed that the range of convergence in feedforward networks is not fixed but varies widely across the regions. For example, between the retina and the lateral geniculate nucleus (LGN) in thalamus, the feedforward pathway relies on a very simple wiring rule, a nearly one-to-one connection between source and target neurons (Usrey et al. 1999), while the wiring from the LGN to the visual cortex follows a much more complicated convergent form (Jin et al. 2011) (Fig. 5a). This implies that the wiring rule of the convergence circuit may be one of the crucial factors for understanding information processing in the visual system. Having shown that each model of the feedforward convergence circuit structure can induce different features of synchronization-specific spike transfer, here we investigated the functional implication of convergence by comparing it to the response of the network from a very small range of convergence (as a model of feedforward wiring from retina to LGN) to large range (from LGN to visual cortex).

To investigate the way how the source signals are transmitted in each convergence condition, we examined the number of source spikes that induce an output spike in the target neuron (Fig. 5b). We implemented the source neuron as a Poisson spike generator with constant mean firing rate of 20 Hz, as in Fig. 1, and then varied the convergence range of the model neural network from 0.5 to 1.25, where ‘1’ is the connection range used in Figs. 2 and 3, and the total connection strength (∑*w*) was kept the same across all the convergence conditions, as before.

We first observed that, in small convergence (range = 0.5), only around two input spikes within 10 ms could induce a target spike in all models (* *p* = 2.2×10^−10^, ** *p* = 2.4×10^−13^, *** *p* = 8.3×10^−14^, n.s. *p* = 1, One-way ANOVA followed by *post hoc* Bonferroni analysis; Fig. 5c), which works as a consistent spike relay (Fig. 5c). On the other hand, as the convergence range became larger, it required a larger number of input spikes within 10 ms to generate an output spike, which was more likely to occur when source spikes were synchronized. This suggests that the network with large convergence would be silent for static input but respond only to synchronized inputs, operating as a spike synchrony detector.

In addition, when the convergence range was small, we found that all three models (UC, GG, UE) operated similarly as a spike relay. However, as the convergence range became larger, we observed a noticeable difference of spike transfer between the models (Fig. 5c). Thus, we found that the spike transfer function of the circuit can vary greatly by both the range and type of convergence wiring in sensory information processing, such as in the visual pathway.

From the perspective of functional implications, a feedforward network with a small range of convergence could be specialized for relaying information, as the thalamic receptive field has a structure similar to that observed in the retina (Usrey et al. 1999). On the other hand, a network with a large convergence could play a role as a coincidence detector. Revisiting our main results, a network with a large convergence could modulate the sensitivity to synchronization, depending on how the synaptic strength is distributed across the connections. As the range increases, the difference between the convergence types becomes important, implying functional diversity in the feedforward network. As observed in experimental studies (Jin et al. 2011; Smith and Häusser 2010), feedforward networks between layers may provide a basic circuit for information processing through convergence wiring.

## Discussion

### Oscillation of firing rate and spike synchronization

Correlations in neural spike activities have been studied extensively in both experimental and theoretical research and a number of studies have reported that synchronized neural spikes might be crucial to information processing in the brain (Buzsáki et al. 1992; Buzsáki and Draguhn 2004; Courtemanche et al. 2003; Donoghue et al. 1998; Engel et al. 2001; Engel and Singer 2001; Fries 2005; Gray and McCormick 1996; Klimesch 1996; Salinas and Sejnowski 2001; Singer and Gray 1995; Varela et al. 2001; Ward 2003; Womelsdorf et al. 2007). In accordance with the view that the spike transfer between neural layers may control the network dynamics, it also has been suggested that the brain may process information selectively through synchronization-dependent modulation of response function or gain of the neural system (Paik et al. 2009; Paik and Glaser 2010). Although the appearance of neural selectivity originated by the convergence between neural layers has been studied in detail (Huerta-Ocampo et al. 2014; Morgan et al. 2016; Wang et al. 2010), there has been little study of whether this synchronization-dependent modulation of spike activity is dependent on the structure of the convergent circuit, or if there is any crucial factor in the convergent structure to control it.

In the current study, first we found that oscillations in the input firing rate could control the synchronization of spike trains. This is consistent with the general idea that various kinds of neural oscillations observed in brain may work as dynamic controllers of neural correlation (Başar et al. 2000; Bastos et al. 2014; Fries et al. 2001; Koepsell et al. 2009; Paik et al. 2009; Paik and Glaser 2010; Salinas and Sejnowski 2001; Uhlhaas and Singer 2010; van Kerkoerle et al. 2014; Wang et al. 2010; Ward 2003). Next, we found that the response of feedforward neural networks is, in general, altered by the level of input synchronization to a certain degree. This suggests that synchronization-dependent neural activity modulation is a generally applicable mechanism for the control of neural response function, even without any changes in the neural circuit such as the number or strength of synaptic connections.

### Synchronization-dependent activity depends on the convergent connection rules

More importantly, we found that synchronization-dependent spike transfer modulation is strongly influenced by the structure of a circuit, in relation to the convergent rule of feedforward wiring. In previous anatomical studies, it was observed that there exist various types of convergent wiring in feedforward neural networks (Felleman and Van Essen 1991; Hubel and Wiesel 1962). For example, in visual systems the feedforward pathway from the retina to the thalamus relies on a very simple wiring rule, close to the one-to-one connection between source and target neurons, while wiring from the thalamus to the visual cortex follows a much more complicated convergent form (Jin et al. 2011; Usrey et al. 1999). This reveals that even in the same feedforward pathway, the structure of feedforward wiring between different layers may have different convergent structures.

Our results suggest that the different convergence structures may work as a different type of synchronization-dependent spike transfer modulator. As we showed in our result, one type of convergence rule may more dynamically modulate the system’s transfer function as the input synchronization increases or decreases, while another type of convergent circuit is relatively insensitive to the change of input correlation. As a result, two types of feedforward circuit may be able to work as a type of information filter or gate, and the brain may develop a different type of convergence structure in different regions of the neural system, as needed for optimal function.

The next question to ask is how these various convergent structures develop in the brain. For example, one possible mechanism that could account for this synaptic wiring could be activity-dependent refinement of neural structure (Butts et al. 2007; Chedotal and Richards 2010; Soto-Treviño et al. 2001). It also might be relevant to a common notion that neurons seek optimal wiring rules that minimize the cost of wiring under particular functional constraints (Bullmore and Sporns 2012; Chen et al. 2006; Chklovskii and Koulakov 2004; Kaiser and Hilgetag 2006; Young and Scannell 1996). In general, it is possible that the optimal wiring rule may vary under different developmental constraints, or by functional structures that should be achieved during development.

In addition, a number of studies suggest that feedback from cortex to subcortical layer, or top-down processing, could contribute to the modulation of feedforward neural activity (Buschman and Miller 2007; Buzsáki and Draguhn 2004; Engel et al. 2001; Moldakarimov et al. 2015; Romei et al. 2010; Saalmann et al. 2007). Our result implies that one possible way to achieve top-down control of the incoming input is to affect the feedforward convergent connection by changing the synaptic weight distribution so that it modulates the synchronization-dependency of the circuits. Further developmental study might be helpful to validate this scenario.

### Various frequency bands of neural oscillation and spike transfer modulation

Among the known brain rhythms, gamma frequency oscillations are considered one of the most interesting features of brain activity and a large number of studies have reported the possible relationship between gamma oscillations and various brain functions (Engel and Singer 2001; Fries 2009; Fries et al. 2007; Paik and Glaser 2010; Sohal et al. 2009; Tiesinga and Sejnowski 2009; Uhlhaas and Singer 2010; Zheng and Colgin 2015). Here we designed our simulation of the oscillating input at 40 Hz, to mimic a gamma-band oscillation. Thus, our results could be interpreted as a mechanism by which the neural system responds to the synchronization induced by gamma band oscillations. This may provide insight into related problems, such as the modulation of sensory information by variation of gamma band power or frequency.

Even though we focused on synchronization at gamma frequency, the mechanism we found here may not be limited to that case. Our findings about the relationship between the feedforward convergence and synchronization could generally be applicable to various conditions of neural networks with gamma or beta frequency oscillations. They might also be applicable to even more complicated cases, such as those in which multiple components of oscillations exist together (*e.g.*, theta and gamma, or beta and gamma). Therefore, our findings here may reveal a general and fundamental mechanism for how the neural system could make use of temporal correlation of inputs to achieve a proper control of its response function.

In summary, we conducted a simulation study on the modulation of information transfer for different level of synchronization of convergent inputs in feedforward networks connected by various convergent rules. Overall, we found that the synchronization-dependent spike transfer strongly depends on the feedforward convergence circuit of a neural network. Our results suggest that, not only the correlation of input spikes, but also the convergent synaptic connectivity patterns in a network, need to be considered to understand the mechanism of information transfer in the brain.

## Electronic supplementary material

ESM 1 (PDF 271 kb)

ESM 2 (PDF 300 kb)
