# Supplementary material for: Synaptic convergence regulates synchronization-dependent spike transfer in feedforward neural networks
Source: J Comput Neurosci. 2017 Sep 12;43(3):189–202. doi: 10.1007/s10827-017-0657-5 (PMC5691111; doi:10.1007/s10827-017-0657-5)
Supplement: Supplementary file 1 — (PDF 271 kb) [file 10827_2017_657_MOESM1_ESM.pdf]

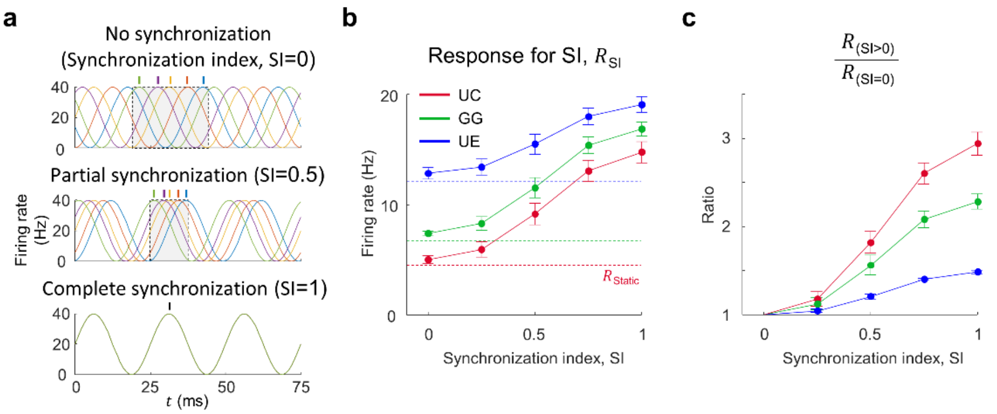

Supplementary Figure S1. Synchronization of oscillation phase in neural inputs.

a Variation of oscillation phase synchronization with strong oscillation. The oscillation phase of each source neuron was randomly selected from a uniform distribution [-(1-SI)π, (1-SI)π] where synchronization index SI, defines the degree of synchronization. For SI = 1, the phase of all neurons is set to '0' (complete synchronization). For SI = 0, the phase of neurons is randomly decided within [- π, π] (no synchronization). Each curve indicates the oscillating phase of a single neuron and the location of the peak is indicated with colored bars. **b** Response (R) of target neurons for different SI with A_f = 1. Response firing rate increases as the level of synchronization increases. Dashed lines indicate the response level for static input. The response at SI = 0 is similar to R_{Static}, but slightly greater in all cases. **c** Response ratio between synchronized (SI > 0) and not synchronized (SI = 0) inputs. As the synchronization index increases, the response increases proportionally. The UC model showed largest ratio in most cases, indicating that the response of which is most sensitive to synchronization level. The averaged ratio at SI = 1 is 2.94 in UC, 2.28 in GG, and 1.48 in UE
