# Supplementary material for: Synaptic convergence regulates synchronization-dependent spike transfer in feedforward neural networks
Source: J Comput Neurosci. 2017 Sep 12;43(3):189–202. doi: 10.1007/s10827-017-0657-5 (PMC5691111; doi:10.1007/s10827-017-0657-5)
Supplement: Supplementary file 2 — (PDF 300 kb) [file 10827_2017_657_MOESM2_ESM.pdf]

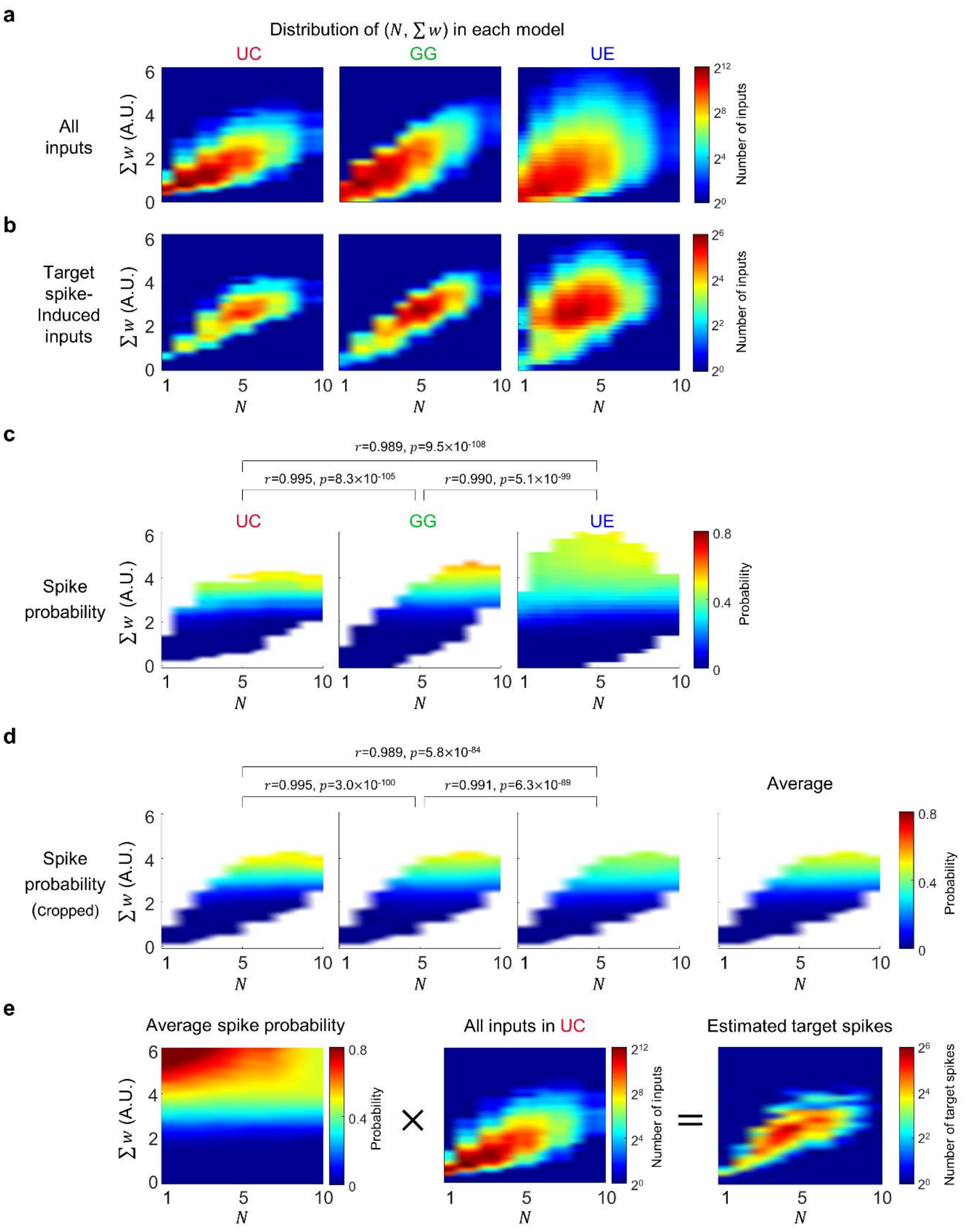

Supplementary Figure S2. Estimation of target cell firing for static input pattern.

a Distribution of input spike patterns of parameter $(N, \sum w)$ in each convergence model for static inputs. **b** Distribution of input spike patterns that induced an output spike in the target neurons. **c** Spike probability distribution for each parameter set $(N, \sum w)$. The conditions that have less than two counts in **a** were excluded to avoid inaccurate estimation by small sample number. Pearson correlation coefficients r between each probability distribution map are shown. Note that all pairs show strong correlation, indicating that the spike probability distribution is fairly similar in all cases.

Only the overlapped regions of valid conditions within a pair were considered during the correlation analysis. **d** Estimation of an average spike probability map from the overlapped area in **c**. **e** Sample estimation of target cell firing Φ for the UC model. First, the complete average spike probability map was estimated from the extrapolation of the average probability map in **d**. Then the number of induced spikes in target cells was estimated by multiplying the average spike probability map (*left*) and the input pattern distribution in **a** (*middle*), and then by summing up the firings for every parameter set $(N, \sum w)$ (*right*)
